# Genetically Engineered Crops: Importance of Diversified Integrated Pest Management for Agricultural Sustainability

**DOI:** 10.3389/fbioe.2019.00024

**Published:** 2019-02-20

**Authors:** Jennifer. A. Anderson, Peter C. Ellsworth, Josias C. Faria, Graham P. Head, Micheal D. K. Owen, Clinton D. Pilcher, Anthony M. Shelton, Michael Meissle

**Affiliations:** ^1^Corteva Agriscience, Agriculture Division of DowDuPont, Johnston, IA, United States; ^2^Department of Entomology, Maricopa Agricultural Center, University of Arizona, Maricopa, AZ, United States; ^3^Empresa Brasileira de Pesquisa Agropecuária (EMBRAPA), Santo Antônio de Goiás, Brazil; ^4^Bayer Crop Science, Chesterfield, MO, United States; ^5^Agronomy Department, Iowa State University, Ames, IA, United States; ^6^Department of Entomology, New York State Agricultural Experiment Station (NYSAES), Cornell University, Geneva, NY, United States; ^7^Research Division Agroecology and Environment, Agroscope, Zurich, Switzerland

**Keywords:** integrated pest management (IPM), genetically engineered (GE) crops, insect resistance management (IRM), integrated weed management (IWM), adoption of technology, sustainability, extension, genetically modified (GM)

## Abstract

As the global population continues to expand, utilizing an integrated approach to pest management will be critically important for food security, agricultural sustainability, and environmental protection. Genetically engineered (GE) crops that provide protection against insects and diseases, or tolerance to herbicides are important tools that complement a diversified integrated pest management (IPM) plan. However, despite the advantages that GE crops may bring for simplifying the approach and improving efficiency of pest and weed control, there are also challenges for successful implementation and sustainable use. This paper considers how several GE traits, including those that confer protection against insects by expression of proteins from *Bacillus thuringiensis* (Bt), traits that confer tolerance to herbicides, and RNAi-based traits that confer resistance to viral pathogens, can be key elements of a diversified IPM plan for several different crops in both developed and developing countries. Additionally, we highlight the importance of community engagement and extension, strong partnership between industry, regulators and farmers, and education and training programs, for achieving long-term success. By leveraging the experiences gained with these GE crops, understanding the limitations of the technology, and considering the successes and failures of GE traits in IPM plans for different crops and regions, we can improve the sustainability and versatility of IPM plans that incorporate these and future technologies.

## Introduction

In 1959, the integrated control concept recognized the many ecological and practical advantages of integrating chemical and biological control strategies for pest management (Stern et al., 1959). The concept of Integrated Pest Management (IPM), a corner stone of Integrated Production (IP), appeared in the 1970's, when it became evident that the overuse of chemical pesticides can have serious negative consequences on the environment and human health. The Food and Agriculture Organization of the United Nations (FAO) defines IPM to be a “careful consideration of all available pest control techniques and subsequent integration of appropriate measures that discourage the development of pest populations and keep pesticides and other interventions to levels that are economically justified and reduce or minimize risks to human health and the environment” (FAO, 2018). Several organizations, including the FAO, the Organization for Economic Co-operation and Development (OECD), and the International Organization for Biological and Integrated Control (IOBC), played a key role in organizing workshops and publishing guidelines related to IPM and IP (Boller et al., 1997, 2004; Wijnands et al., 2012; FAO, 2018; OECD, 2018). IPM is now recognized as a desirable standard for plant protection internationally (e.g., FAO, European Union Directive 2009/128/EC, US Food Quality Protection Act of 1996).

The foundation of an IPM approach is the use of indirect (preventive) crop protection practices, which rely on an understanding of the environment, crop, pest and natural enemy biology, and use of optimized farming practices to manage pests. This includes selection of appropriate crop cultivars for the region, management of soil, nutrients, and water, utilization of sustainable pest suppression practices, as well as implementation of practices that foster the abundance and diversity of beneficial species, such as natural enemies, decomposers, and pollinators. As part of the IPM approach, key pests are closely monitored, and defined intervention thresholds for pest damage or presence are used to indicate when a direct (responsive) crop protection practice is warranted. When required to supplement the preventive practices, the consideration and integration of a broadly diversified set of biological, biotechnical and physical control tactics (e.g., release of natural enemies, pheromone traps or release of sterile insects, and utilization of nets or tillage, respectively) are key to formulation of a diversified, durable, yet flexible IPM strategy that meets social requirements for economic, environmental and human health protection. When pesticides need to be applied, products that are selective are preferred over broad spectrum pesticides. In addition, it is recommended that pesticides are applied with appropriate equipment, optimal dosage, and best timing (Boller et al., 2004; Ervin and Jussaume, 2014; Owen, 2016).

Host plant resistance, whether developed through conventional breeding or through genetic engineering (GE), is a cornerstone of IPM and is a complementary tool to other pest management practices. GE crops have been grown on increasing areas since 1996, reaching 190 million hectares in 2016 globally (ISAAA, 2017). Most GE crops provide tolerance to herbicides (e.g., glyphosate, glufosinate-ammonium, dicamba, or 2-4 D), protection against lepidopteran and/or coleopteran pests, or a combination of both traits. For example, herbicide tolerance (HT) traits that confer glyphosate resistance are available in soybean, maize, canola, cotton, sugar beet and alfalfa, while insect protection, which to date has predominantly been conferred by insecticidal proteins derived from *Bacillus thuringiensis* (Bt), is available in cotton, soybean (lepidopteran pests), and maize (lepidopteran and coleopteran pests). Eggplant in Bangladesh has also contained a Bt trait for a lepidopteran pest since 2014. Additional HT traits that provide tolerance to other herbicidal active ingredients (e.g., isoxaflutole) and other insect active traits (using RNAi, other non-Bt insecticidal proteins, etc.) are being developed to expand the portfolio of GE crops (ISAAA, 2019).

While GE crops may offer additional tools to complement IPM programs and improve their sustainability, economics, and social factors (for example, how one grower's pest management decisions affect surrounding growers and community; Ervin and Jussaume, 2014; Ervin and Frisvold, 2016), an understanding of the characteristics of the crop, the introduced GE trait(s), the crop production system, and the socioeconomic context is critical to successfully integrating GE crops into IPM systems (Meissle, 2016). Current developments in IPM, insect resistance management (IRM) and managing herbicide-resistant weeds were highlighted in a recent symposium organized within the 14th International Symposium on the Biosafety of Genetically Modified Organisms, in Guadalajara, Mexico. Over a series of presentations and a panel discussion, the principles of IPM, the role of socio-economic factors, comprehensive extension to grower communities, and regulations in IPM adoption, and the benefits of using GE crops in an integrated system to improve sustainability were discussed. We present in this paper several case studies where GE crops have been used to manage insects, weeds and diseases and, using these case studies, we highlight the opportunities and challenges for successfully integrating GE crops into an IPM approach in both developed and developing countries. Our examples include GE crops and traits where experience has been gained over many years (e.g., Bt crops, HT crops), new GE plants that have just entered commercial production (Bt eggplant), and GE plants that have not yet been planted commercially (virus resistant common bean).

## Opportunities and Challenges for Using Bt Crops in IPM

Over the past 30 years, traits have progressed from single events with one mode of action against one insect order, to pyramided and stacked events containing multiple modes of action against the same or different pest orders, respectively. GE crops have also progressed from insect protection traits expressing proteins from Bt to new traits based on RNAi or expressing proteins from non-Bt sources (ISAAA, 2019). There are many widely accepted benefits of using GE crops for insect control, including the ability to reduce the use of less effective and/or less environmentally friendly insecticides, high specificity toward pests, and a more convenient insect pest management strategy for growers (Brookes and Barfoot, 2013, 2016). An additional benefit seen in some systems, such as with Bt maize in the US (Hutchison et al., 2010; Dively et al., 2018) and Bt cotton in China (Wu et al., 2008) and the US (Carrière et al., 2003), has been area-wide suppression of key target pests that has reduced pest pressure and input costs for both growers adopting Bt crops and non-adopters in the same area. Nevertheless, there remain several challenges for sustainable use of this technology and successful implementation in an IPM approach for many Bt crops and regions.

One of the biggest challenges for sustainable use of the technology is the evolution of resistance. Over-reliance on Bt crops without appropriate IRM or IPM practices has led to a growing number of cases of target pest resistance (Gassmann et al., 2014; Tabashnik and Carrière, 2017). Examples include field-evolved resistance to Cry1Ab-expressing maize in the African stalk borer, *Busseola fusca* (Fuller) (Lep.: Noctuidae), in South Africa (Van Rensburg, 2007); resistance to Cry1F-expressing maize in the fall armyworm, *Spodoptera frugiperda* (J. E. Smith) (Fuller) (Lep.: Noctuidae), in Puerto Rico, Brazil and Argentina, and the mainland US (Storer et al., 2010; Farias et al., 2014; Huang et al., 2014); resistance to Cry1Ac-expressing cotton in the pink bollworm, *Pectinophora gossypiella* (Saunders) (Lep.: Gelechiidae), in India (Dhurua and Gujar, 2011); and resistance to Cry3Bb1-expressing maize in the western corn rootworm, *Diabrotica virgifera virgifera* LeConte (Col.: Chrysomelidae), in the US (Gassmann et al., 2011, 2014).

To address the risk of insect resistance, IRM programs have been proactively implemented wherever Bt crops have been commercialized, with these programs being mandatory in some countries including the USA, Canada, Australia, the EU, the Philippines and South Africa (Matten et al., 2008). Central to these IRM programs is the concept of a “refuge,” which is an area of plants (typically of the crop of interest) that do not contain any Bt protein and thereby support the production of Bt-susceptible insects (Gould, 1998; Gould et al., 2016). Refuges represent a short-term cost to growers because they incur greater pest damage and require additional management, and thus refuge adoption by growers is generally much higher in countries where IRM is a regulatory requirement e.g., Australia, Canada and the US. The Australian cotton industry represents one success story for adoption of IRM. In the 1990s, Australian cotton-growers faced near catastrophic levels of Lepidoptera resistance to insecticides, which almost led to the end of the cotton industry (Roush, 1998; Roush et al., 1998; Fitt, 2003; Wilson et al., 2018). High awareness of the need for IRM by growers, the availability of different refuge options, and appropriate education and training has resulted in refuge adoption that is consistently near 100% in Australia. Similarly, intensive education together with auditing of growers have helped to maintain high levels of refuge adoption in other countries like Canada (91%) [Canadian Corn Pest Coalition (CCPC), 2018] and, to a lesser extent, the US Corn Belt (68–72%) [Agricultural Biotechnology Stewardship Technical Committee (ABSTC), 2016]. In areas where IRM is not a requirement, disincentives are very high, or growers are not as aware of the costs of resistance, it remains a challenge to educate growers, demonstrate the long-term value of the refuge strategy, and identify other tools to balance the short-term costs. The absence of robust IRM programs can have major consequences; for example, in all the cases of field-evolved resistance described above, one of the primary causes was determined to be low refuge compliance (Tabashnik et al., 2013). Examples of countries where IRM management programs are not mandated include Argentina, Brazil, and China (Wu, 2007; Choudhary and Gaur, 2008). In addition to the lack of refuge compliance, other factors contributing to the evolution of resistance include less-than-high-dose technologies and diverse pest complexes. Overall, regulating IRM and integrating GE crops within the context of a larger IPM plan can help to ensure success, particularly with technologies that are not high dose, but will not be sufficient to do so without extension that leads to broad stakeholder support. Demonstrating the value of IRM within the context of IPM, for example showcasing how GE crops and refugia can better support populations of natural enemies (Lu et al., 2012), or positioning IPM strategies as solutions to greater pest damage in refuges and for non-adopters of GE crops, are important benefits to highlight to promote an integrated approach. For example, insect predator and aphid populations in Bt cotton fields in northern China were assessed over 20 years, from 1990 to 2010, to test the hypothesis that Bt crops can promote biocontrol services at a landscape level (Lu et al., 2012). Results from this study showed that Bt cotton fields with reduced insecticide application supported higher predator populations and decreased aphid abundance. This work supports the hypothesis that widespread adoption of Bt cotton may promote landscape level benefits due to increased generalist predator abundance, and reinforces how IPM strategies that utilize Bt crops and reducing insecticide application can achieve more effective biological control (Romeis et al., 2018).

An additional challenge associated with Bt crops can result if there is a pest shift (i.e., increased prominence of a secondary pest that was collaterally or incidentally controlled by broad-spectrum insecticides but is not controlled by the selective GE trait). For example, in China, widespread adoption of Bt cotton, and the associated decreased use of chemical insecticides, has led to increased abundance of mirid bugs (Hemiptera: Miridae) in some fields (Lu et al., 2010). Any time a primary pest is significantly reduced or eliminated by a technology including a GE trait, there exists the possibility that replacement inputs or other ecological factors will result in a pest shift that may require additional crop protection inputs. If those additional inputs are selective, the overall gains made by growers may still be very positive and IPM is strengthened (Naranjo and Ellsworth, 2009a,b; Ellsworth et al., 2017). However, when new inputs are broad-spectrum, the benefits of adopting the GE trait could be significantly diminished both because of the new input costs and lost opportunities for environmental and human health benefits. A well-structured IPM approach should balance the use of one technology with other complementary approaches and avoid relying on only one solution for pest control. Genetic engineering is not a “silver bullet” for all problems and an agricultural production system will not automatically become a durable IPM strategy just by adding GE technology or, for that matter, host plant resistance developed through conventional means. Therefore, understanding the challenges for each crop, pest complex and region and acknowledging the limitations of GE crops is important for education, training and development of robust IPM strategies for future crops and traits.

## IPM of cotton in Arizona and Mexico

Cotton production in the desert Southwest U.S. has been historically challenged by the presence of several key insect pests and a wide array of secondary pests. By the early 1990s, the boll weevil, *Anthonomus grandis* Boheman (Col.: Curculionidae), had been successfully eradicated from Arizona through a combination of areawide cultural and chemical practices. At about the same time, the invasive whitefly, a cryptospecies of *Bemisia tabaci* (Gennadius) (Hem.: Aleyrodidae) [ = *B. argentifolii* Bellows and Perring], arrived in southern California and Arizona with devastating consequences and established as a key pest of cotton, vegetables and melons thereafter (Ellsworth and Martinez-Carrillo, 2001). This leaf-sucking pest remains today as the number one threat to cotton quality due to their deposits of copious sugary excrement on fiber (Ellsworth et al., 2017). A mirid bug, *Lygus hesperus* (Knight) (Hem.: Miridae), feeds directly on reproductive structures (especially buds and flowers), reducing the number of fruiting sites on the plant and threatening yield production. Another key pest is a boll attacking lepidopteran, the pink bollworm *P. gossypiella*, which is challenging to control because of its cryptic feeding habits inside bolls.

During the first half of the 1990s, insect pest management was dependent on the routine deployment of broad-spectrum chemical controls, such as organophosphates, carbamates and cyclodienes, and pyrethroid mixtures. Resistance and costly secondary outbreaks with mites (Acari), aphids (Hem.: Aphididae), saltmarsh caterpillars [*Estigmene acrea* (Drury), Lep.: Erebidae], cotton leaf perforators (*Bucculatrix thurberiella* Busck, Lep.: Bucculatricidae) or cabbage loopers [*Trichoplusia ni* (Hübner), Lep.: Noctuidae] were common. Foliar spray use was intensive with statewide averages of 10–13 sprays per season (Naranjo and Ellsworth, 2009b).

The introduction of Cry1Ac-containing Bt cotton varieties in 1996 helped to usher in a new era of selective pest control. This trait effectively conferred immunity in cotton to the pink bollworm. Coincidentally, that same year saw the introduction of two selective insect growth regulators (IGRs) for the control of whiteflies. Immediate reductions in foliar insecticide use resulted, though control of *Lygus* bugs still required broad-spectrum insecticides. The success of the GE cotton cultivars was marked by exceptional adoption rates, peaking in 2008 with more than 98% of acreage in Bt cotton after the initiation of a grower-organized pink bollworm eradication campaign (Naranjo and Ellsworth, 2010; Tabashnik et al., 2010). With the introduction of flonicamid, a feeding inhibitor, as the first selective chemical control of *Lygus* bugs in 2006, growers had opportunities to manage all three key pests without the use of broad-spectrum chemistries. The result was an increased role for conservation biological control and a step-change reduction in the use of foliar insecticide (Naranjo and Ellsworth, 2009a; Naranjo et al., 2015; Ellsworth et al., 2017). Starting in 2006 and continuing to this day, Arizona cotton growers spray insecticides, on average, 2.0 ± 0.2 times for all arthropod pests with virtually no sprays for lepidopterans. And, the vast majority of Lygus and whitefly sprays are made with beneficial friendly, fully selective insecticides (Ellsworth, personal communication).

With each new technological innovation (i.e., Bt cotton, selective whitefly IGRs, *Lygus* feeding inhibitor), there was a concomitant need for extensive education, outreach, and extension to growers and their pest managers. Innovations span a continuum of hard technologies (typically complete products like improved seeds, traits, chemicals) to soft technologies (knowledge-based, human-mediated techniques like how to sample and implement thresholds, and IPM strategies). Broad extension support provided by the U.S. Cooperative Extension System is organized federally, within states, and locally within counties to educate, train and facilitate technology transfer to stakeholders. While hard technologies are often technically easy-to-use, their proper deployment depends on accompanying soft technologies that include important translational research and extension adaptation and implementation on a local scale.

The success of the Arizona cotton IPM strategy with Bt cotton as the cornerstone building block of the management system (Ellsworth and Martinez-Carrillo, 2001) was not possible without the significant, ongoing, and progressive inputs from extension as an organized force of mission-oriented research and engaged outreach. Working with Mexican cotton growers immediately across the U.S. border from Arizona and California provided a unique opportunity to examine a counterfactual in a very similar ecoregion and production environment, and largest cotton production region of that country. Their access to most of the hard technologies was contemporaneous to when Arizona cotton growers were adopting them, but there was no analog to Cooperative Extension in Mexico. While Bt cotton was adopted in this region of Mexico at a relatively high rate, growers were still spraying many more times than their Arizona counterparts and exclusively with broad-spectrum insecticides (e.g., methamidophos and many other organophosphates, endosulfan, pyrethroids). Funded by a 17-month grant from US-EPA, an Arizona team conducted an intensive extension campaign in Mexico including grower education, workshops, seminars, demonstrations, and grower participatory trials and validation research (Ellsworth, personal communication). As a result, in 2012 alone, growers decreased their spraying by 31–40%, their insecticide costs by 34% and reduced the use of broad-spectrum insecticides by 23–86% for a savings of over $1.6 million. This lends support to the conclusion that GE crops like Bt cotton or any other hard technology are very dependent on the set of adaptive research and strategic solutions that constitute soft technologies (especially IPM), and further that Cooperative Extension or an analog is key to the transfer of both hard and soft technologies simultaneously.

The Arizona cotton IPM strategy has cumulatively saved growers over $500 million since 1996 in yield protection and control costs ($274/ha/year), while preventing over 25 million pounds of active ingredient from being used in the environment (Ellsworth et al., 2017). While the uptake of Bt cotton and other selective technologies was critical to enabling greater reliance on natural controls like conservation biological control, the key to success was ongoing, progressive development of soft technologies that built-out the IPM strategy and the continued investments in engaged outreach and grower education to support proper integration and compatibility of practices. As such, GE crops are a powerful, selective, and therefore enabling tactical elements of IPM that, when properly integrated and stewarded, can help maximize benefits to stakeholder while minimizing downside risks.

As already noted, structured refuges usually of the same host plant are critical components to the durability of Bt traits in GE plant systems. However, functional refuges can be supplied through novel means, the deployment of sterile insect technique (SIT) and/or pheromone-based mating disruption. Arizona cotton grower organizations in partnership with industry, university research and extension, and state and federal regulatory agencies embarked on an eradication program that permitted growers to plant up to 100% of their cotton to Bt cultivars without planted refuges starting in 2006. Refuges were supplied by targeted and proportional releases of sterile male pink bollworm moths over Bt and non-Bt fields throughout Arizona and mating disruption (Naranjo and Ellsworth, 2010; Tabashnik et al., 2010, 2012). Supported by cultural and other measures, this eradication campaign extended throughout all infested states of the U.S. and northern Mexico, resulting in the rare achievement of eradication of the pink bollworm and recent lifting of related cotton quarantines of U.S. cotton in October of 2018 (USDA, 2018).

**Figure 1:**
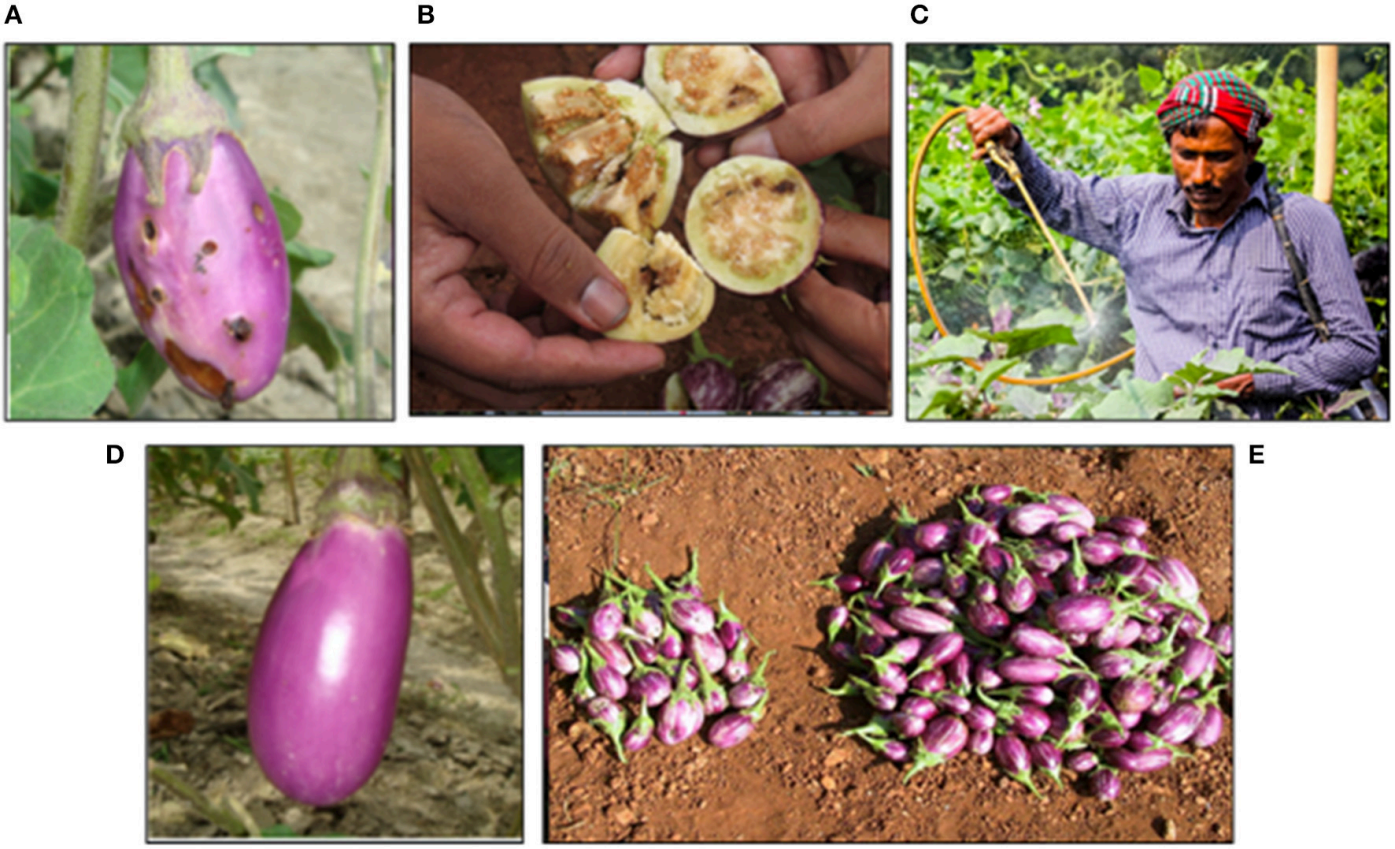
**(A)** Eggplant, *Solanum melongena* L. (also known as brinjal) damaged by the eggplant fruit and shoot borer (EFSB), *Leucinodes orbonalis* Guenée (Lep.: Crambidae). **(B)** EFSB burrowing in the fruit of an eggplant. **(C)** Farmer Shahjahan spraying pesticide without appropriate personal protective equipment (i.e., gloves, mask, eye protection, etc.) in his brinjal field. **(D)** Bt brinjal line, Uttara, grown as part of a field trial in Bangladesh to demonstrate efficacy. **(E)** Bt brinjal (right) compared to non-Bt brinjal (left) as an example to demonstrate potential increased yield.

Enabled and strengthened by the proper integration of hard technologies like GE crops, the Arizona cotton IPM strategy entailed the development, integration, and extension of no fewer than 15 other tactical building blocks (see Figure 1 in Ellsworth and Martinez-Carrillo, 2001), many rooted in knowledge-based, soft technologies (e.g., sampling plans, action thresholds, resistance management). The central, foundational tactic of conservation biological control enabled by selective technological inputs is responsible for at least 42% of the economic gains made by Arizona cotton growers (Ellsworth et al., 2017). Much of the balance of these gains (58%) are due to the hard technologies *per se*, including Bt cotton inclusive of their actual grower costs. This remarkable stability and durability of this IPM system likely emboldened growers to mount the eradication campaign and contributed in large measure to this successful outcome. Refuges, structured between 1996 and 2005 and in the form of SIT starting in 2006, and resistance management goals have also benefited by the remarkable gains in conservation biological control. Furthermore, biological control was potentially important to supporting the extirpation of the pink bollworm, the primary target pest species of Bt cotton in Arizona.

## IPM of Bt eggplant in Bangladesh and the Philippines

While the advent of GE crops was a transformative success story in agriculture for maize, cotton and soybean, the use of Bt crops has almost entirely been limited to these large acreage commodity crops (Shelton et al., 2017). Research and development of GE technology for “minor” crops have not been as prominent. This is unfortunate because this group of crops includes fruits and vegetables, both of which are needed for a balanced, nutritious diet and for diversified farm income. Furthermore, fruits and vegetables tend to be heavily treated with insecticides because of their diverse insect complexes, high market value, and strict cosmetic requirements (Shelton et al., 2008), resulting in what is often referred to as the produce paradox (Palumbo and Castle, 2009). The role of GE crops in an IPM strategy for many minor crops remains largely untapped but the example of eggplant demonstrates the potential benefits.

Eggplant, *Solanum melongena* L. (also known as brinjal in India and Bangladesh, and talong in the Philippines) is one of the most important, inexpensive and popular vegetable crops grown and consumed in Asia. The biggest constraint to eggplant production throughout Asia is the chronic and widespread infestation by the eggplant fruit and shoot borer (EFSB), *Leucinodes orbonalis* Guenée (Lep.: Crambidae) (Figures 1A,B). Infestation levels may exceed 90% and the yield loss has been estimated up to 86% in Bangladesh (Ali et al., 1980). It has been reported that 98% of Bangladeshi farmers relied solely on insecticide applications to control EFSB (Karim, 2004) and farmers spray insecticide nearly every day or every alternate day with as many as 84 applications during a 6–7 month cropping season (BARI, 1994). Such heavy reliance on insecticides, including broad-spectrum organophosphate, carbamate and pyrethroid insecticides, has been implicated in negative effects on human health and the environment (Dasgupta et al., 2005). Similarly, in the Philippines, damage by EFSB can result in yield loss of 80% and control relies primarily on frequent applications of insecticides (Francisco, 2009). Unfortunately, in resource poor areas in Bangladesh and the Philippines, these pesticides are often applied without the appropriate protective equipment, resulting in high and prolonged exposures to farmers (Figure 1C). Due to the high potential for pest damage, current lack of alternative tools or strategies for managing this pest, and high economic value of this crop, there is a great opportunity for leveraging GE technology as a tool for an IPM strategy. Furthermore, because EFSB is a close relative of the European corn borer which was so successfully controlled by Bt maize, it was suggested that Bt eggplant might also be an appropriate management strategy for EFSB.

The development of Bt eggplant began in 2000 by the India-based Maharashtra Hybrid Seed Company (Mahyco) under a partnership with Monsanto Company, using a *cry1Ac* gene that had already been widely used in Bt cotton in India. The *cry1Ac* gene expresses the Cry1Ac protein, which confers protection against specific lepidopteran pests, including EFSB. Research and development of the Bt eggplant included efficacy trials, and control of EFSB was demonstrated in contained greenhouse trials (Choudhary and Gaur, 2008). A partnership was developed with Mahyco, Cornell University, the United States Agency for International Development (USAID) and public sector partners in India, Bangladesh and the Philippines under the Agricultural Biotechnology Support Program II (ABSPII) in 2003. Bangladesh was the first country to approve cultivation of Bt brinjal and, on 22 January 2014, Bt seedlings were distributed among 20 farmers in four districts in Bangladesh. Due to the clear benefits of Bt brinjal for EFSB control, adoption of the GE technology has increased each year. In 2017, more than 6,000 small-scale, resource-poor farmers in Bangladesh grew Bt brinjal on their farms. In 2018, adoption increased to more than 27,000 farmers (Shelton et al., 2018). In fact, this estimate may even be higher because the distributed seed is open-pollinated and growers can save seed from the previous year.

Studies have shown that Bt brinjal provides nearly complete control of EFSB and dramatically reduces insecticide use, providing tremendous economic, health, and environmental benefits to farmers (Shelton et al., 2018) (Figures 1D,E). Preliminary socioeconomic studies indicate that Bt brinjal farmers have a six-fold increase in income, compared to non-Bt brinjal farmers. As with any effective host plant resistance technology for insects, the reduced need to spray for the key pest (EFSB) will have cascading effects in the agro-ecosystem and affect IPM tactics. For example, other tactics will be needed to control the complex of “sucking insect pests,” but this can be done through use of more selective insecticides or through enhanced biological control through conservation of natural enemies. As shown with other cropping systems (see examples of Bt maize in Brazil and cotton in Arizona and Mexico), use of Bt plants has allowed natural enemies to play a more prominent role for control of primary and secondary pests, such as sucking insects. Studies in the Philippines have already shown important natural enemies are conserved when using Bt eggplant (Navasero et al., 2016) and many studies have shown that conservation of natural enemies through the use of Bt plants can help them control secondary pests (e.g., Tian et al., 2015). Furthermore, studies have also shown that natural enemies can contribute to delaying the evolution of Bt resistance in the key pest species (Liu et al., 2014), a win-win situation for farmers.

In Bangladesh, the Minister of Agriculture has been an outspoken and strong supporter of biotechnology and this has been an essential factor in its adoption (Shelton et al., 2017). Meanwhile, the USAID partnership program is trying to move forward in the Philippines by helping them develop and submit a strong regulatory dossier. However, in India, where research on Bt eggplant first originated and where the Genetic Engineering Committee of India approved its commercialization in 2009, Bt eggplant is still not grown because of political pressure on the Minister of the Environment and Forests resulting in a moratorium that is still in place today (Shelton, 2010).

Besides the regulatory challenges for Bt eggplant, there are other significant challenges and foremost is good stewardship. The USAID partnership program works with the Bangladesh Agricultural Research Institute (BARI) as its implementing partner. In February 2018, scientific and technical project staff conducted a 4-day workshop and training program at BARI on gene equivalency and maintaining line purity (Cornell University, 2018; Hossain and Menon, 2018). Even before the seed is delivered, it is vital that the farmer receives adequate training on this new technology. Prior to the first release of Bt brinjal, BARI conducted training and continues to emphasize that Bt brinjal needs to be treated for other insects and diseases, and non-Bt brinjal should be planted as border rows (refuge) to delay the evolution of resistance. Monitoring for adherence to seed quality and refuge planting by farmers is critical for the sustainability of Bt brinjal. Furthermore, monitoring for changes in susceptibility of EFSB to Cry1Ac in the field is an essential component of tracking sustainability. To date, there are limited data available on baseline susceptibility, however additional studies are underway. Likewise, plans need to be developed to incorporate an additional Bt gene into lines to enhance their durability. This should be done before resistance to Cry1Ac occurs (Zhao et al., 2005) and will require regulatory adroitness and new licensing agreements with the technology provider of the dual gene event. Other strategies including pheromonal disruption are being investigated in other brinjal projects in SE Asia and, if successful and economically feasible, can be incorporated in a Bt brinjal IPM program as a complementary tactic. Meanwhile, if resistance does occur, the government will need to implement contingency plans for how to control EFSB. Unfortunately, these strategies are costlier, more labor intensive and less effective (Talekar, 2002).

Proper stewardship is a challenge in any country, but even more so in a developing country like Bangladesh that does not have experience with GE field crops and with a crop like Bt brinjal for which the farmers can save the seed. Farmer training is a vital component of the program and needs to emphasize IPM concepts to ensure the durability of this valuable product. But if the challenges associated with Bt brinjal can be overcome and sustainable solutions implemented, Bt eggplant represents a great advance in the farmer's ability to manage ESFB damage in this crop as part of an IPM approach. It also points the way forward to using biotechnology for minor crops in developing and industrial countries for control of major pests, while reducing the use of traditional pesticides.

## IPM of Bt Maize in Brazil

Maize is an important crop grown in Brazil and *S. frugiperda* (fall armyworm) is the major maize pest (Blanco et al., 2016). Pest populations have intensified over the years due, in part, to growers planting maize during a second growing season. This creates a “green bridge” that provides continuous host plants and allows *S. frugiperda* to complete up to 8–10 generations a year on maize (Storer et al., 2012). Prior to GE maize, Brazilian growers primarily controlled *S. frugiperda* with insecticides. Instead of scouting and use of economic thresholds, growers typically sprayed prophylactically every 1–2 weeks due to the polyphagous feeding habits, migratory abilities from field to field, and multiple overlapping immigrations into a field during early corn growth (Cruz, 1995). Growers have also historically increased spray rates and volumes to improve larval mortality once the larvae move to the whorl. Conversely, growers' use of aerial spray applications over larger fields has resulted in reduced spray coverage due to lower volumes delivered, which also likely decreased the effective dose against *S. frugiperda* larvae. Control practices like these in combination with the challenging biology of *S. frugiperda* has led to rapid resistance evolution to many insecticides in Brazil (Diez-Rodeiguez and Omoto, 2001).

Recent introductions of GE technology (2008–2010), targeting *S. frugiperda*, in Brazil have provided levels of crop protection in maize not previously realized by Brazilian growers. The GE maize produces various Bt proteins (including Cry1Ab, Cry1F, Cry1A.105, Cry2Ab, Vip3Aa) that are toxic (at varying levels) to *S. frugiperda* larvae upon ingestion of plant tissue. Although the high risk of resistance evolution to Bt was recognized at the time of commercialization and IRM recommendations were provided by industry, resistance has quickly evolved to multiple Bt proteins (particularly Cry1A and Cry1F) within 3–4 years (Farias et al., 2014; Omoto et al., 2016).

Rapid resistance evolution to Bt proteins is thought to be a result of the deployment of these products without meeting key assumptions for the high-dose/refuge resistance management strategy. Three assumptions should be met for this strategy: (1) recessive inheritance of resistance in pest species; (2) low initial resistance allele frequency; and (3) abundant refuges of non-Bt host plants near Bt crops promoting random mating (Tabashnik et al., 2013). GE crops deployed in Brazil to date have all violated at least one of these important prerequisites (Tabashnik et al., 2013). Low refuge compliance in Brazil is one common issue faced by all GE Bt products and, as discussed earlier, Brazil is one country where IRM is not required through regulation. Minimal industry and grower adoption of refuges contributed to the accelerated resistance evolution observed with *S. frugiperda*. Resistance allele frequency against Cry1A and Cry1F proteins also appears to have been relatively high with *S. frugiperda* populations leading to quick evolution of resistance (Farias et al., 2016; Omoto et al., 2016). Finally, proteins like the Cry1As and Cry1F are known not to be high-dose against *S. frugiperda* (Vélez et al., 2016).

One proposed resistance management solution to these Bt resistance problems has been the introduction of Bt pyramids to affected geographies like Brazil. GE pyramid products express at least two proteins that are effective against the same target insect. Due to cross-resistance among similar Bt proteins, the effectiveness of the pyramid strategy in Brazil as a resistance management tool has been limited thus far (Bernardi et al., 2015). Cross-crop resistance is another concern in diverse crop landscapes where multiple crops share similar Bt proteins. Research results suggest that if cross-crop resistance occurs among different Bt crops, landscapes like Brazil where corn, cotton, and soybean share similar Bt proteins, the selection period for cross-crop insects will be extended and thus accelerate resistance evolution (Yang et al., 2016). Therefore, rapid resistance evolution with pests like *S. frugiperda*, is likely linked to multiple factors described in this case study.

Resistance management has a limited likelihood of success if GE products like those described above are not placed into a well-understood IPM framework capable of sustaining the value of these technologies. The potential utility and contribution of IPM tactics including cultural and biological controls need to be better understood. The industry has developed several initiatives to drive the implementation of refuges and best management practices (BMPs) with growers. Industry alignment meetings led by the Insecticide Resistance Action Committee (IRAC) were initiated in 2015 to develop BMPs for maize, soybean and cotton farmers. Industry also developed several pilot programs with growers to educate and provide incentives for adopting refuge, though these have resulted in minimal uptake to this point. Although research continues to refine management tactics to use with GE and non-GE refuge crops, tropical geographies like Brazil that harbor pests like *S. frugiperda* will challenge IPM and IRM strategies. Socioeconomic factors should be combined with agricultural systems knowledge to develop an industry framework that drives adoption of key IPM and IRM practices. In addition, regulation that requires critical resistance management tactics like the planting of refuges should be pursued. Until either or both of these approaches are further developed, deploying new GE technologies in countries like Brazil should proceed with caution.

## IPM of Bean Golden Mosaic Virus in Common Bean in Brazil

Common bean (*Phaseolus vulgaris* L.) is an important staple food in Brazil and other countries in Latin America. Similar to brinjal, common bean is an orphan crop that can utilize GE technology to complement the IPM approach for managing bean golden mosaic virus (BGMV). BGMV is the causal agent of the most destructive viral disease of common beans in Brazil. It is efficiently vectored by the whitefly, *B. tabaci*, which is also a significant insect pest for this and several other crops, especially in tropical areas. BGMV causes stunted growth, yellowing and flower abortion, and high yield losses (Anderson et al., 2016). Traditional pest control tactics for the insect vector are limited to chemical pesticide application, and overuse of pesticides on common beans is a common problem leading to environmental effects and insect resistance problems (Bonfim et al., 2007).

GE common bean was modified using RNAi technology to develop a BGMV resistant variety by Brazilian Agricultural Research Corporation (Embrapa) (Bonfim et al., 2007; de Faria et al., 2016). BGMV resistant common bean was granted commercial approval by Brazil in 2011 [Comissão Técnica Nacional de Biossegurança (CTNBio), 2011] and was registered and protected as cultivar BRS FC401 RMD by the Brazilian Ministry of Agriculture, Livestock and Food Supply in 2016 (Souza et al., 2018). GE common bean offers an opportunity to farmers to control this viral pathogen without chemicals. However, there remain several key challenges for successful integration of this technology into a sustainable IPM plan. Following regulatory approval, the current challenge is to successfully insert this GE trait into commercial varieties that are optimized for the different regions (Souza et al., 2018). Additionally, IPM and farm management practices are being optimized and farmer training is being offered to ensure sustainable use and durability of the trait. For example, management strategies including implementing a whitefly host-free period (elimination of hosts for both virus and whitefly), designating sentinel areas (where common bean fields are planted early in the season to screen for the presence and abundance of viruliferous whitefly populations), and optimizing planting time and chemical control practices are all valuable components of the emerging IPM plan. These tactics are important to reduce damage by whitefly due to direct feeding as well as deposition of honeydew on which mold fungi can grow and reduce photosynthesis. Additionally, it is important to reduce areawide pressure of whitefly as a disease vector because, while BGMV is the most devastating virus, it is not the only whitefly transmitted virus to common beans (Brown et al., 2015). New geminiviruses [*Macroptilium* yellow spot virus—MaYSV; Soybean chlorotic spot virus—SoCSV; and *Macroptilium* yellow vein virus—MaYVV (Sobrinho et al., 2014)] are a threat to common beans in Northeastern Brazil, and the flexivirus, Cowpea mild mottle virus, is a destructive disease of common beans (de Faria et al., 2016).

Building professional capacity through farmer training, and developing an alert system to quickly identify if a threshold for pest population or viral pathogen load is being exceeded will also be critical to success. Because the GE common bean varieties have not yet been commercialized, this work to optimize management practices and increase farmer training is being conducted with growers on small plots (up to a half hectare). There have been encouraging results with implementing whitefly host-free periods and using an alert system to evaluate the real need for chemical control. Going forward, the use of whitefly monitoring/reporting system and the sentinel areas will help growers to make the correct decision about whether to grow common beans or switch to an alternative crop to maximize income with lower risks of crop losses. GE common bean with resistance to BGMV will help to diversify the tool box for IPM in Brazil, and an integrated approach to pest management of whitefly is essential for achieving agricultural and environmental sustainability, food security and grower profitability. IPM practices (including whitefly monitoring, sentinel areas, pest free periods, etc.) must be continued and leveraged to enable decision-making and successful integration of a sustainable IPM plan.

## Integrated Weed Management (IWM) With Herbicide Tolerant Crops

Weed management strategies have not changed greatly in the last five decades. Despite the adoption of GE crops with HT traits, weed management arguably is still largely, if not exclusively, based on herbicides. HT crops have many advantages, and the benefits of being able to use herbicides that would cause unacceptable phytotoxicity to a crop (e.g., glyphosate) are clear. However, to date, HT traits are largely limited to conferring tolerance to a few herbicidal active ingredients, and a small subset of commercial commodity crops. Therefore, many opportunities to expand the portfolio of HT traits in crops with this technology remain, considering that the availability of herbicides for use in high value crops such as fresh vegetables is limited. If HT traits were available in some high value crops, the effectiveness of weed control would improve greatly, the costs of weed control would decline and the quality of the crop would increase (Gianessi, 2013).

Despite the unprecedented success of HT technology for weed management, successful implementation and sustainability of this technology presents many challenges, including the evolution of herbicide resistance in key weed species. Success of HT crops is seen as increased simplicity of weed management, improved time management and reduced costs; farmers as a result, became increasingly unwilling to adopt integrated weed management (IWM) practices including the need for multiple herbicidal modes of action to address evolved herbicide resistances in weeds (Frisvold et al., 2009; Norsworthy et al., 2012). These challenges highlight the need for diverse, well-designed and proper IWM plans. It is important to recognize that herbicide resistance evolution is not necessarily a reflection on the cultivation of HT crops. Rather, herbicide resistance has been a prominent problem for agriculture since the beginning of herbicide use (Heap, 2019). The issue of evolved herbicide resistance in key weeds reflects the fact that herbicides have been the principle tactic for weed control for more than 45 years and the inclusion of alternate strategies for weed management has declined steadily over the same period of time (Jussaume and Ervin, 2016; Owen, 2016). For example, glyphosate has been applied on the majority of row crop acres in the US for more than two decades. While there are many reasons and justifications for this approach including improved time management and efficiency, reduced costs for weed control, as well as increased effectiveness, simplicity and convenience, the ecologically narrow focus of one approach unsurprisingly resulted in rapid and widespread evolved resistance to glyphosate within important weed species such as *Amaranthus tuberculatus* J. D. Sauer, *A. palmeri* S. Wats, and *Conyza canadensis* (L.) Cronquist (Owen et al., 2015). This clearly demonstrates why weed management in row crops is not sustainable if based primarily on a single herbicide.

Because herbicides will likely continue to play a significant role in weed management in the future, designing robust management plans for weeds will be important for sustainability of HT crops. Unfortunately, strategies associated with IPM for insect pests are often not applicable for weeds (Owen, 2013, 2016). For example, concepts such as action thresholds for insect damage have no utility in weed management, given the growth plasticity of weeds, the high amount of seeds produced, and the long life of seeds in the soil seedbank. In fact, often the decision to allow weeds to remain uncontrolled because the population density is below a theoretic economic injury level at one point in time will result in greater weed problems in the future. Similarly, IPM programs for insects and diseases are typically developed around one pest species; whereas for weed communities found in crop fields, many species, each with different ecological characteristics and management requirements need to be considered. For example, different weed species affect the crop at different times of the growing season which complicates the timing of control tactics. Furthermore, many weeds have numerous germination events, each of which requires control while insect pests tend to have fewer emergence events that simplifies the timing of control tactics. Finally, with weeds, the pest targets are closer morphologically, phenologically, physiologically and biologically to crops than insect or diseases, which presents additional challenges and limits the flexibility of control tactics. Nevertheless, the need for sustainable and durable tactics for weed control is important. The development of an IWM strategy, which includes diverse tactics other than herbicides for weed control, complements the concept and foundational approach of IPM programs developed for other pest complexes (Swanton and Weise, 1991; Swanton et al., 2008; Owen, 2016).

Diverse IWM strategies include, but are not limited to, cultural and biological tactics that can supplement mechanical and herbicide-based weed management approaches and will be important components of successful weed management programs in the future (Meissle, 2016; Owen, 2016). Examples of diverse strategies that supplement an herbicide-based weed management plan include, but are not limited to harvest weed seed destruction (Walsh et al., 2018) and more diverse crop rotations employed in a crop system (Blackshaw et al., 2008). Related to seed destruction for example, Walsh et al. (2018) illustrated the successes of reducing the weed seedbank, and describes several tactics that can be used during crop harvest that destroy weed seeds thus improving weed management efforts in the future. Similarly, Blackshaw et al. (2008) demonstrated the positive effects of using diverse crop rotations [in this case, GE canola (*Brassica napus* L.) and forages such as alfalfa (*Medicago sativa* L.)] for reducing weed population densities and improving overall weed management in a cereal-based crop production system. While it may be simpler to depend on a few weed management practices, the key to sustainability will be for all entities involved in weed management, private, commercial and government, to consider more diverse weed management approaches. For example, Iowa, a key US state for maize and soybean production, currently is developing a state Pest Resistance Management Plan established by an inclusive committee that represented all agricultural groups and sponsored by the Iowa Department of Agriculture and Land Stewardship and the Iowa State University College of Agriculture and Life Sciences (Iowa Pest Resistance Management Program, 2018). The plan provides guidelines for establishing management programs for herbicide-resistant weeds and consists of pilot projects demonstrating community-based weed management. However, the specifics of the conceptualized diverse and community-based management plans for herbicide-resistant weeds have yet to be developed and implemented.

Herbicide-resistant weeds represent a “wicked” problem, in that there is no single strategy for weed management and new technological advances alone will not resolve the issue (Ervin and Jussaume, 2014; Ervin and Frisvold, 2016). Herbicide resistant weeds are very mobile within an agricultural community, and while local solutions should be adaptable to an individual grower's needs, they must align with the broader weed management goals at a landscape or regional level (Ervin and Jussaume, 2014). Confounding the effort to manage those weeds are multiple herbicide resistances in a majority of key weed populations (Owen et al., 2015). While some farmers may recognize the importance of community involvement with regard to herbicide resistance management, some feel that any efforts put forward will be for naught, as their neighbors will not participate in the effort (Doohan et al., 2010; Barrett et al., 2017). As previously discussed by Davis and Frisvold (2017), “The efficacy of any pesticide is an exhaustible resource that can be depleted over time. For decades, the dominant paradigm—that weed mobility is low relative to insect pests and pathogens, that there is an ample stream of new weed control technologies in the commercial pipeline, and that technology suppliers have sufficient economic incentives and market power to delay resistance supported a laissez faire approach to herbicide resistance management. Earlier market data bolstered the belief that private incentives and voluntary actions were sufficient to manage resistance. Yet, there has been a steady growth in resistant weeds, while no new commercial herbicide modes of action (MOAs) have been discovered in 30 years” (Davis and Frisvold, 2017). Unless there is a community-based effort put forth to manage herbicide resistance that goes beyond using herbicides, it is unlikely that any effort will be successful. Therefore, while GE crops may offer great opportunities for weed control in agriculture, there remains a critical need to adopt diverse tactics other than herbicides to manage resistant weeds and to reduce the risk of herbicide resistance evolution where it has not yet become a problem.

## Discussion

The goal of an IPM strategy is to support the sustainable production of high quality crops while minimizing environmental impacts attributable to pests or pest management practices. While the benefits of using an IPM approach are evident, as outlined in the case studies above, implementation of IPM can be very challenging for several reasons (Box 1) (Meissle, 2016). One common theme among the case studies presented is that a successful IPM or IWM strategy leverages a diversified approach. GE crops should not be viewed as a “silver bullet,” and while their success may seem like an infallible solution to control pests in the short run, durability and sustainable use requires a long-term vision. As can be seen based on the many years of experience using Bt and HT traits, insects and weeds will inevitably evolve resistance over time. Part of the goal of the IPM plan is to diversify the approaches to pest management, and limit the dependence on one single technology. Just as it is crucial for IPM practices (including whitefly monitoring, sentinel areas, pest free periods, etc.) to be continued for whitefly control in common beans in Brazil, it is equally critical that comparable IPM practices are developed, optimized and maintained for all crops and pests. Knowledge and understanding of the technology, pest, crop, region, alternative tools and even social contexts are critical for the success of an IPM plan, because if there is insufficient understanding of the technology and how best to integrate it into an IPM system, the durability of the technology may fail. In addition, if there is not adequate training and engagement of farmers to recognize the short- and long-term benefits of the management plan, the technology may fail due to lack of compliance. Incentives may be needed to gain producer compliance with best management and resistance management requirements, and often farmer training is needed to demonstrate the short-term and long-term benefits of implementing a sustainable approach.

Box 1 **Challenges and solutions for successful implementation of an integrated pest management (IPM) approach.**

**Table:** 

**Challenges**	**Solutions**
IPM is knowledge-driven. Information about the biology and ecology of pests and natural enemies, development and testing of appropriate tools and strategies, and training of farmers to use these tools appropriately is needed for successful deployment.	A strong partnership is needed between industry, regulators and farmers, with an emphasis on farmer training. Training should emphasize IPM concepts, benefits and limitations of tools and pest management strategies, and the importance of compliance. Innovative solutions and best management practices aimed at sustainability must continue to be developed, adapted and implemented on a local scale. Individual and areawide benefits and challenges need to be identified, discussed, and promoted early in the development and deployment processes of GE technologies When changes in management strategies are needed, a communication plan and intensive education program are needed to transfer knowledge.
IPM plans need to serve environmental, human health, and social goals while being economically beneficial to the producer.	Incentives are needed for the implementation of an IPM strategy that maintains the ecological infrastructure, facilitates the implementation of crop rotations, and supports the application of environmentally friendly pest control systems to gain compliance. The IPM approach in each region should consider the cultural and socio-economic contexts. Farmer training and education can put the short-term costs into context with the long-term benefits of implementing a sustainable approach. An industry and government partnership for IRM regulation ensures compliance to IRM and IPM practices.
GE technology is often perceived as a “silver bullet.”	GE crops are implemented within a comprehensive IPM strategy. A range of efficient and economically feasible options including, but not limited to the GE crops, needs to be available for a given production system and region. Understanding that pest shifts may occur, integrated solutions should not only address the current pest spectrum, but should also consider the possibility of pest shifts. GE traits should complement a broader IPM strategy filled with companion and compatible selective tactics for all the other pest challenges in the system. Farmer training and education can transfer knowledge, leverage the experiences gained with GE crops, and allow for a better understanding of the benefits and limitations of each technology.
Regulatory restrictions, high costs for developers, and long approval timelines for GE crops can restrict the availability of options for farmers and greatly reduce and delay benefits to society.	A science-based approach for the safety assessment and regulation of GE crops is needed. Public discussions need to move from a focus on GE technology in general to a focus on cropping systems, their problems, and potential solutions, including GE crops. Collaboration and communication among consumers, academics, industry, and government, that leverage the experience and familiarity gained with the cultivation of GM crops over the last twenty years, is needed to transfer knowledge, and allow for a better understanding of the benefits and limitations of GE crops.

Likewise, training stakeholders about how best to integrate and use GE crops in their existing agricultural system is critical. While industry tends to focus on discovery, research and development and promoting the value of GE traits, there is a huge responsibility for institutions (e.g., government, public, and private) to make the investments necessary to develop the systems that consider not only the technical solutions possible, but also the cultural and socio-economic contexts. As suggested by Stern et al., “one reason for the apparent incompatibility of biological and chemical control is our failure to recognize that the control of arthropod populations is a complex ecological problem. This leads to the error of imposing insecticides on the ecosystem, rather than fitting them into it” (Stern et al., 1959). On the other hand, if the technology and tactics are fit to the existing system, and appropriate training is provided to stakeholders, there is a much higher chance of success and sustainability over time.

Because of these (and other) challenges for successful implementation of an IPM approach, pest control based on broad spectrum chemicals is often perceived as the easier, more economic, and most efficient short term approach used for pest management in large-scale farming operations. To promote continued research, expand implementation, and highlight the value of using an IPM strategy, a joint effort among governments, label organizations, growers, grower associations, and the seed and pesticide industries is critically needed. Most of the major successes in gaining grower support for resistance management over the past 50 years were preceded by pest resistance-related economic failures and the solutions involved a strong partnership between industry, regulators and farmers. Innovative solutions and BMPs aimed at sustainability must continue to be developed in particular for crops and regions where there is high resistance risk (e.g., tropical production systems), or grower adoption of resistance management requirements has failed.

The benefits of a successful IPM strategy, including reduced application of broad spectrum chemical pesticides, more durable pest management in ecologically balanced crop production systems, and reduced risks to human health and the environment, are clear. Sustainable, eco-rational IPM strategies rely on a diversified portfolio of tactics, of which GE crops represent a valuable tool. By leveraging the experiences gained with GE crops, understanding the limitations of the technology, and considering the successes of GE traits in IPM plans for different crops and regions, we can enhance the durability and versatility of IPM plans for future crops.

## Author Contributions

All authors listed have made a substantial contribution to the conceptualization and drafting of the manuscript. All authors contributed to manuscript revision, read and approved the submitted version.

### Conflict of Interest Statement

During the writing of this paper, JA and CP were employed by Corteva Agriscience™, Agriculture Division of DowDuPont, GH was employed by Monsanto Company, JF was employed by Empresa Brasileira de Pesquisa Agropecuária (EMBRAPA), and MM was employed by Agroscope. The remaining authors declare that the research was conducted in the absence of any commercial or financial relationships that could be construed as a potential conflict of interest.
